# Plasmodium falciparum and P. malariae: infection rates in the population of Northern Imbo Plain, Burundi

**DOI:** 10.24248/eahrj.v4i2.643

**Published:** 2020-11-26

**Authors:** Hermann Nimpaye, Desiré Nisubire, Joseph Nyandwi

**Affiliations:** a University of Burundi, Faculty of Medecine, Department of Laboratories, Burundi; b University of Burundi, Faculty of Medecine, Department of internal Medecine, Burundi

## Abstract

**Background:**

Burundi is cited among countries where malaria remains endemic. Notably, malaria is highly endemic in Imbo region, a lowland lying astride Lake Tanganyika. Among key malaria riposte interventions includes the promotion of Long-Lasting Insecticidal Nets (LLINs), but its incidence rate has not reduced. In this paper, we present the distribution of malaria species in 2 settings within Imbo region by accounting for the seasonal variations and the mostly infected populations.

**Methods:**

The study was conducted from 2 Health Care Centres of Murambi and Rugombo in Cibitoke District, Northern Burundi. Blood samples were collected on blood slides and the samples were used to confirm the presence of malaria parasites by microscopy.

**Results:**

The study observed an average malaria parasite prevalence of 32.5% across the selected site. Majority of patients 459(95.2%) were infected by P. falciparum while 8(1.7%) patients were infected by P. malariae. Patients from Murambi were more infected than those from Rugombo. P. falciparum was the most highly prevalent specie in the 2 localities. High prevalence was observed in children aged between 2 and 5 years. Among older participants P. falciparum still predominated and mixed infections were rather the least prevalent.

**Conclusion:**

This study showed that P. falciparum and P. malariae are the most parasites involved in malaria morbidity in North Imbo region. The transmission of P. falciparum was observed year-round. Patients in Murambi are most exposed to malaria infections than those in Rugombo. Further research at large scale including entomological studies is required to better understand the relationship between Entomological Inoculation Rates (EIR) and malaria transmission levels in this setting.

## BACKGROUND

In most Sub-Saharan African countries, malaria remains a public health threat. In 2018, the world malaria report estimated an incidence of 218 million cases which translated into nearly 405,000 deaths. The Sub-Saharan Africa (SSA) accounted for about 93% of malaria-related deaths. In this Subcontinent, malaria accounts for 10% of global deaths and up to 50% of hospital admissions.^1^ Markedly, children below 5 years and pregnant women are the most vulnerable to malaria.^2^ For instance, in 2018, children below 5 represented 67 % of annual malaria-related death toll.^1^

Until today, Burundi is cited among countries where malaria remains endemic.^3^ The country undergoes seasonal malaria epidemics, causing thousands of deaths each year.^4,5^ Notably, malaria is highly endemic in Imbo region, a lowland lying astride Lake Tanganyika.^6^ In this region, rice growing has significantly contributed to malaria endemicity as this constitutes a favourable breeding site for mosquitoes.^7^ Among key malaria riposte interventions include the promotion of Long-Lasting Insecticidal Nets (LLINs) use which the Government has noticeably invested in for more than a decade.^8^ However, despite enormous efforts to control malaria by the Government and other players, its incidence rate has not reduced.^9^ It is against this statement that we conducted a study to shed more light on the current malaria transmission and endemicity rates in Imbo region. In this paper, we present the distribution of malaria species in two settings within Imbo Region by accounting for the seasonal variations and the mostly infected populations.

## METHODS

### Study Area

**FIGURE 1.**
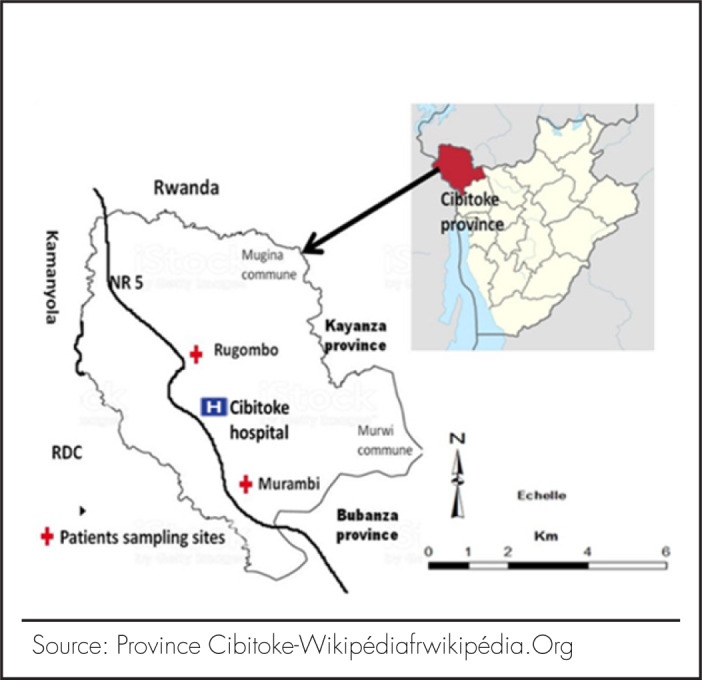
Study areas in Cibitoke Province, Burundi

Source: Province Cibitoke-Wikipédiafrwikipédia.Org

The study was conducted in two sub-settings namely Murambi and Rugombo in Northern Imbo lowland. Murambi is located on 02° 08’’ Latitude South and 29° 04’’ Longitude East and Rugombo on 02° 54’’ Latitude South and 29° 08’’ Longitude East. These settings are a Sub region of Cibitoke Province, Rugombo Commune.^10^ Unlike most parts of the country, the study setting experiences high temperatures, typically around 24 degrees Celsius year-round. In addition to high malaria-related reported deaths, the rationale behind the selection of the study setting includes the lack of recent data to inform policymakers. The most recent malaria data from this region was published in 1984 as can be seen in Figure 1. The study setting encompasses 3 Health Centres and one Hospital. Bowing to our capacity, the study was conducted in two conviniently selected Health Centres of Murambi and Rugombo.

### Study Type

We conducted a cross-sectional study using data collected on a period of 12 months from January to December 2014.

### Simple Size

The study was conducted on a sample of 1,482 patients from whom malaria Rapid Test Diagnosis (RTD) was requested by the treating nurse.

### Data Collection

From all patients undergoing malaria rapid test, an additional blood sample was collected for the purpose of the study. The sample was used to confirm the presence of malaria parasites by using Ethanol and Giemsa (ARCHEM^®^: AlliwinEliixer Organic) and to further determine the type of parasite under microscopy (Olympus Corporation, Tokyo, Japan). Data was collected twice a month and for a period of 12 months. No data collectors were recruited as the study required laboratory skills. Only researchers were allowed to read malaria blood samples and capture data into an Excel Spreadsheet.

### Data Management

Collected data was entered into Excel spreadsheet and imported into STATISTIX PC DOSVersion 2.0 Copyright (C), 1987, NH Analytical Software and Fischer Test (California, USA). Data entry was crosschecked by two data capturers to ensure consistency and track missing values. Any missing value was recollected immediately by referring to the samples. In the first instance, we calculated malaria prevalence followed by the distribution of malaria species across the study settings. In the second time, patients were disaggregated by age category and malaria prevalence by type of species and calculated for each age category. Finally, a test of proportions was used to seize significant differences between malaria prevalence and again by age category.

### Ethical Consideration

The study obtained ethical clearance from the National Ethics Committee. Furthermore, the study obtained special permission from the Provincial Health and District Health Officers respectively. All participants signed individual informed consent forms. The bio bank was shredded after data analysis to avoid unethical future uses. Also, each participant was provided with an identification number for anonymous reasons.

## RESULTS

### Plasmodia Species Distribution

As shown in Table 1, of 1,482 patients, 482(33.4%) were infected by at least one of Plasmodia species with an average parasite index of 32.5%. Majority of patients 459(95.2%) were infected by *P. falciparum* while only 8(1.7%) and 15(3.1 %) were diagnosed with *P.malariae* and mixed infections, respectively.

**TABLE 1:** Prevalence of P. falciparum and P.malari

	Study sites	Murambi (%)	Rugombo (%)	F	p-value	OR	CI
Species							
P. falciparum		220 (35.2)	239 (27.8)	5.0	0.0	1.4	1.0-2.0
P. malariae		2 (0.3)	6 (0.7)	0.5	0.3	-	
P.f; P.m		2 (0.3)	3 (0.3)	0.6	0.5	-	
P. m; Sch. P. m		2 (0.3)	5 (0.5)	0.7	0.4	-	
Total		227 (36.3)	255 (29.7)	8.0	0.001	3.2	1.6-6.5

SD: Signifcative difference; NSD: Non Signifcative Difference; P. f : Plasmodium falciparum; P. m: Plasmodium malariae; Schiz. P. m: Schizonte de P. malariae

Comparison of results from Murambi 227(36.3%) and Rugombo 255(29.7%) showed a statistically significant difference (F=8.0, p= .0 and OR=3.2 [1.6-6.5]). On the one hand, *P. falciparum* was the most highly prevalent specie in the 2 localities with 220(35.2%) and 239(27.88%) infected patients in Murambi and Rugombo respectively. Again, the difference between proportions was signifi-cant (F=5.0, p= .0 and OR=1.4[1.0-2.0]). On the other hand*, P. malariae* was less distributed with only 2(0.3 %) and 6(0.7%) of cases in Murambi and Rugombo respectively. Furthermore, there was no difference between the localities (F=0.9, p=.3).

Similar to *P. malariae*, we found rare mixed infections with P. falciparum and *P. malariae*; these cases accounted for only 2(0.3%) and 3(0.3%) in Murambi and Rugombo. We did not find evidence for a statistical difference between the areas. (F=0.4, p=.5).

Equally, accounting for parasite evolution stages (schizont-associated trophozoites), less than 1% of patients were infected with *P. malariae*. In Murambi, the study detected 2 cases (0.3%) and 5 patients in Rugombo (0.5%). We did not detect statically significant difference between the 2 proportions (F=0.5, p=.4).

### Plasmodia Species Distribution According to Age Stages

In Table 2, we present results of *P. falciparum* and *P. malariae* as well as mixed infections by age of participants. The table highlights that the vast magnitude of infections occured before the age of 24 months. In fact, 20(21.9%) of P. falciparum infections were found among participants aged below 6 months and 142(35.8%) among those aged between 7 and 23 months. The least represented infections were P. malariae with 2(0.5%) patients and mixed infections representing 3(0.5%) patients.

**TABLE 2:** Malaria Prevalence by Age Category

Age range	0–6 months	7–23 months	2–5 years	6–10 years	11–15 years	>15 years	Total
Infections							
P. falciparum	20 (21.9)	142 (35.9)	208 (39.1)	36 (43.3)	14 (29.8)	40 (12)	460
P. malariae	0 (0.0)	2 (0.5)	5 (0.9)	1 (1.2)	0 (0.0)	0 (0.0)	8
Mixed-infections	0 (0.0)	3 (0.5)	5 (0.7)	0 (0.0)	2 (2.1)	4 (1.2)	14
Total	20 (21.9)	147 (37.1)	218 (40.9)	37 (44.5)	16 (34)	44 (1.2)	482

Among children aged between 2 and 5 years, 208(39.1%) carried P. falciparum. In this age category, only 5(0.9%) were infected by P. malariae and another 5(0.7%) by various mixed infections. For older children, those aged above 5 and below 10 years, nearly half (43.37%) were infected by P. falciparum. In this age trench, only 1 patient (1.21%) was diagnosed with P.malariae. Above the age of 10 and below 15 years, almost one-third (29.8%) of patients had P. falciparum while only 2(2.1%) patients carried mixed infections. Among older participants (above the age of 15 years), still P. falciparum predominated with 12% of patients and mixed infections were rather least prevalent (1.2%).

### Seasonal Malaria Transmission

**FIGURE 2.**
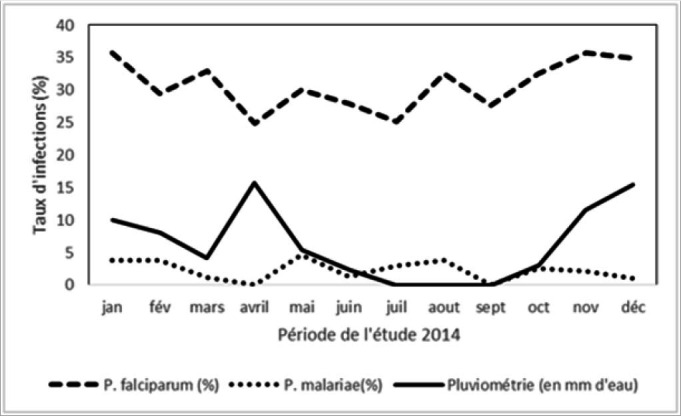
Malaria Transmission According to Seasons

As seen in Figure 2, there was transmission of P. falciparum throughout the year with seasonal variations. In contrary, P. malariae is only seasonal as there were no cases for a period of 6 months in the year (from April until September). To highlight noticeable malaria transmission differences, for instance, a peak of P. falciparum cases was observed in April (24.3%) corresponding to heavy rain season (14.3 mm of water). Conversely, during dry season, from July to September, we observed a peak of P. malariae, with 3.4% cases.

### Malaria Distribution among Children Aged Between 2 and 9 Years Old

**FIGURE 3.**
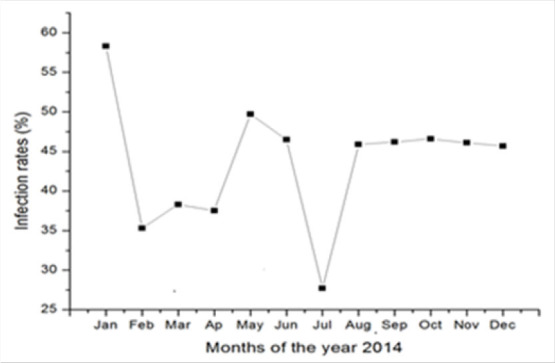
Annual Malaria Transmission among Children Aged 2-9 Years Old

The annual rate transmission of *P. falciparum* parasites in children aged between 2 and 9 years varied from 27.7% to 58.3%. The peak was observed in January (58.3%) with fewer cases in July (27.7%). Variations in malaria cases did not exhibit important magnitude for the rest of the year. (Figure 3).

## DISCUSSION

The aim of this study was to determine and characterise transmission and endemicity levels of malaria in 2 localities (Murambi and Rugombo) in Northern Imbo lowland in western Burundi.

Overall, *P. falciparum* was the most prevalent specie found in the two study settings. Similar results were found in the preceding studies. For example, in 1991, Barutwanayo M and Coosemans M et al., reported that *P. falciparum* was associated with the high morbidity due to malaria observed in the Northern Region of Imbo.^6^

Further, the study exhibited that malaria is highly prevalent in Murambi than in Rugombo. Plasmodic index of *P. falciparum* in Murambi was higher than in Rugombo. Our results corroborate those obtained by previous authors^11^ including Coosmans who showed that plasmodic index is relatively low in Rugombo locality (16%) than in Ndava (27%) located not far from Murambi on Ruhwa-Bujumbura pathway.^8^ All these results showed that the rate of malaria transmission was higher in savannah than in urban locality. To support the above statement, previous studies also reported that malaria was highly transmitted in rural regions in Niger^12^ and other studies reported similar results in the savannah regions of West and Central Africa.^13^

In our study, in comparison with children (0-6 months), adolescents and adults (from the age of 11 years onwards); findings showed that infants aged from 7 months up to 10 years are highly vulnerable to malaria. Boudin C and Robert V et al., reported that children below 6 months are protected by maternal antibodies^14^ and this ephemeral premonition decreases with age and may be depleted when babies are 2 years old unless maintained by anopheline infective bites.^15^ Anti-malaria premonition, which increases with age may be generally established after adolescence among populations being regularly exposed to infective mosquito bites.^16^

In our study, rain seasons predicted significant rise in P. falciparum transmission. Such a correlation is highly suggestive as rain seasons are associated with the transformation of marshes into irrigated croplands which is at the origin of a high malaria prevalence.^15,16^ This could explain the high malaria morbidity in the study population given the fact that Murambi lies aside Muhira River and several rice irrigation canals. To add on that, irrigated rice farming practiced in both Rugombo and Murambi contributes to the creation of potential breeding sites for vector multiplication and the maintenance of malaria transmission and its evolution under the hyper-endemic mode.^5,8^ Studies in Burkina Faso^17^ and that by Nanga-Eboko in Cameroon^19^ showed that the entomological inoculation rates are positively correlated with increased rainfall intensity. Also, P. falciparum and P. malariae cases occur during dry season, suggesting the presence of potential breeding sites for vector multiplication. There were potential water collections in irrigated rice farms and relevant stream water^20^. Very few P. falciparum and P. malaria species in mixed infections were reported in this study. Such findings can be associated with the low of P. malariae transmission as observed during the entire study period. Our findings stream together with finding from previous studies.^4,8^

### Strengths and Limitations

This study, which informed stakeholders and decision makers on the prevalence and parasite distribution of malaria in Imbo region, used data from a significantly big sample to ensure generalisability. In addition, blood samples were taken and captured by lab technicians, which helped to ensure data accuracy and consistency. However, we did not confront blood samples with rapid diagnostic tests for better case detection. Also, despite the ability to inform on associations, cross-sectional designs do not allow causality inference.

## CONCLUSION

This study showed that P. falciparum and P. malariae are the most parasites involved in malaria morbidity in North Imbo region. The transmission of P. falciparum was observed year-round. Patients in Murambi are more exposed to malaria infections than those in Rugombo. High malaria-related morbidity was observed in children between 2 and 5 years old. In the study setting, malaria transmission was stable with a trend to turn into hyper endemic. We recommend further investigations at large scale including entomological studies to better understand the relationship between Entomological Inoculation Rates (EIR) and malaria transmission levels in this setting. New preventive measures such as environmental interventions, campaigns for better LLINs use, and the promoting of research would contribute to reduction of malaria incidences
